# Electrode positions, transformation coordinates for ECG reconstruction from S-ICD vectors

**DOI:** 10.1016/j.dib.2017.02.041

**Published:** 2017-02-22

**Authors:** David G. Wilson, Peter L. Cronbach, D. Panfilo, Saul E. Greenhut, Berthold P. Stegemann, John M. Morgan

**Affiliations:** a University of Southampton, UK; b University Hospital of Southampton NHS Foundation Trust, UK; c Medtronic Bakken Research Center, The Netherlands; d Medtronic Minneapolis, USA

## Abstract

The article contains data pertaining to the reconstruction of an 8-lead ECG from 2 subcutaneous implantable cardioverter defibrillator vectors. The location of electrodes on the precordium required for the data collection are detailed; the flow chart for patient selection and exclusion is shown; the summary data of the root mean square error (RMSE) (in microvolts) and Pearson *r* for the ECG transformation all cases and the pearson correlation for all the leads measured and reconstructed leads are also shown. Detailed background, methodology and discussion can be found in the linked research article.

**Specifications Table**

**Table:** 

Subject area	*Medicine*
More specific subject area	*Cardiology*
Type of data	*Table and figures*
How data was acquired	*Electrocardiographic recordings*
Data format	*Analyzed*
Experimental factors	*Acquisition of surface electrocardiograms*
Experimental features	*Deriving transformation coefficients and comparing derived leads to measured leads*
Data source location	*Southampton*, *United Kingdom*
Data accessibility	*Data is in the article*

**Value of the data**

• The conversion of two subcutaneous implantable cardioverter defibrillator vectors into an 8 lead surface ECG, has never been achieved before.
• These data provide detailed insight into how this was achieved.
• The summary data of the root mean square error (RMSE) (in microvolts) and Pearson *r* for the ECG transformation all cases and the pearson correlation for all leads would be invaluable to other investigators to compare their results to.

## 1 Data

**Fig. 1:**
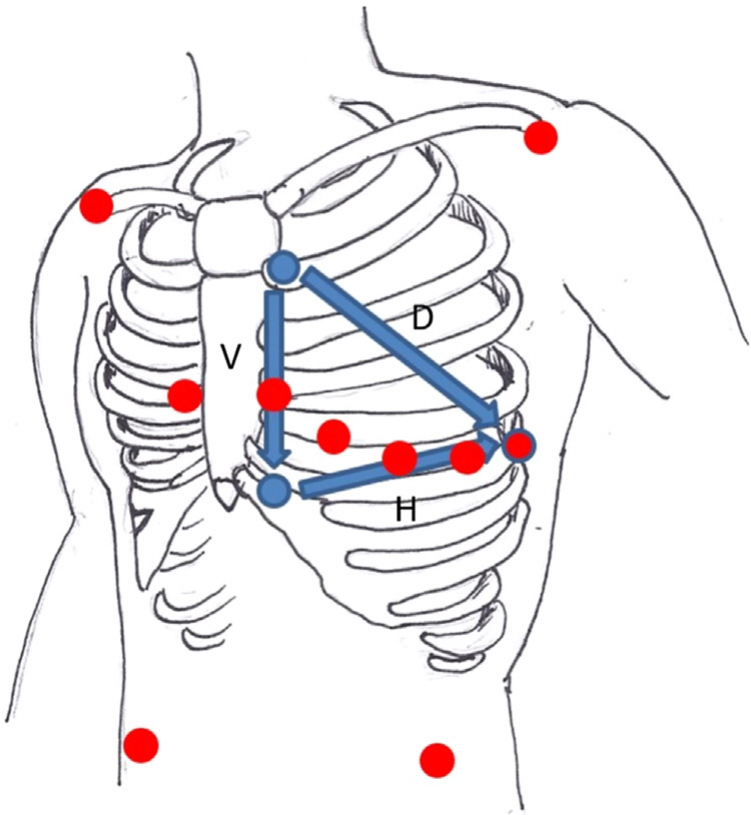
Illustration of the Mason–Likar 12-lead electrode arrangement (red dots) and three study S-ICD electrodes placed in the conventional S-ICD electrode positions, (blue dots) and the location of the horizontal (H), vertical (V) and diagonal (D) vectors.

**Fig. 2:**
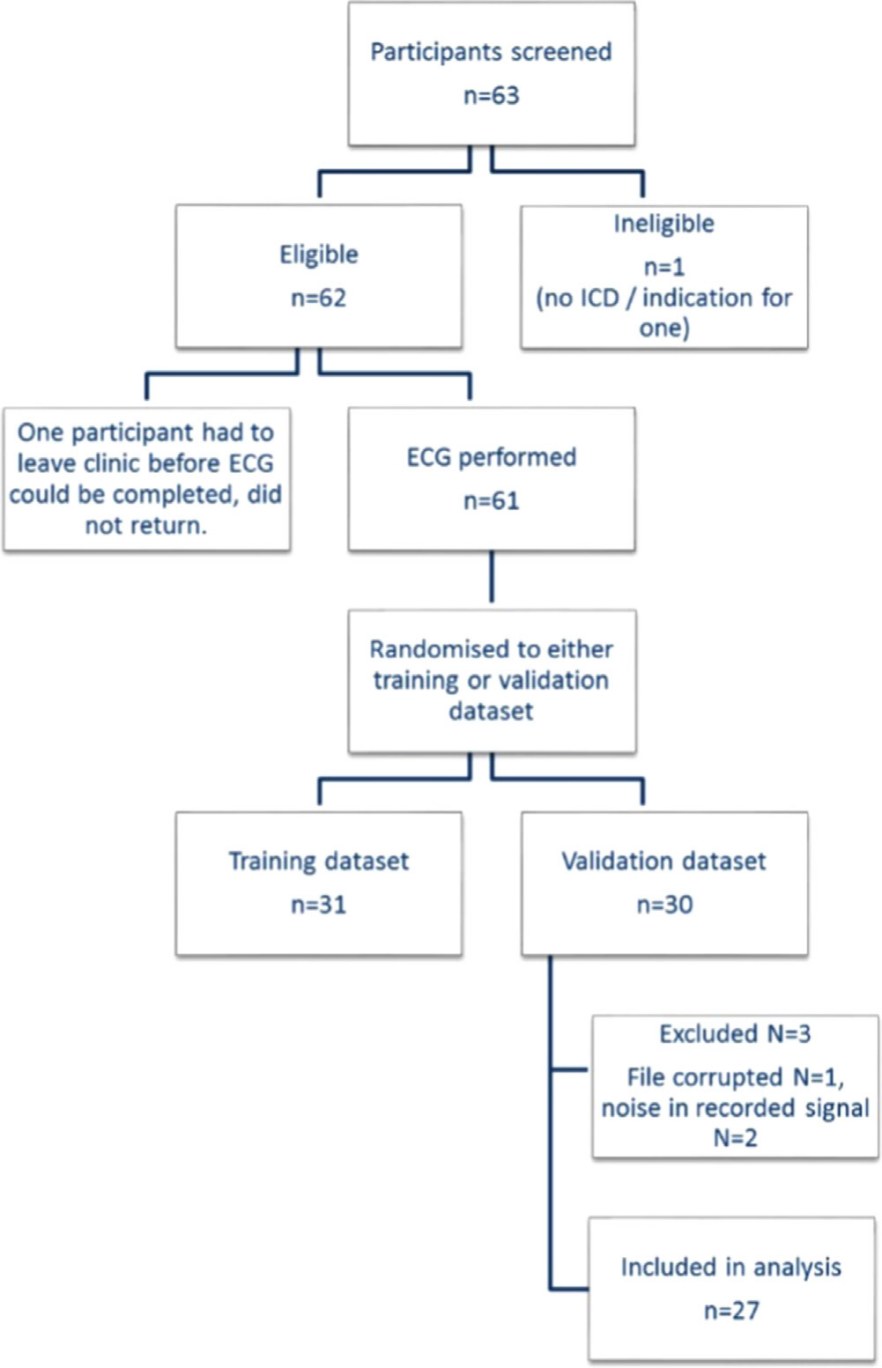
Flow chart for selection of participants.

The data provided in this data article compliment the original research article that described the transformation of two subcutaneous implantable cardioverter defibrillator vectors into an 8-lead surface ECG. Fig. 1 illustrate the location of the electrodes on the precordium used during data acquisition and the Fig. 2 illustrates the flow of subjects through the study. In Table 1 the outcome of the comparison of the originally measured and derived (transformed) leads are summarized using root mean square error and Pearson correlation coefficient. Table 2 demonstrates the Pearson correlation coefficients for all patients and all leads.

## 2 Experimental design, materials and methods

### 2.1 Materials and methods

#### 2.1.1 Study population

All patients with an ICD indication aged at 18 years and over attending the ICD clinic at Southampton General Hospital were eligible for this study. Informed, written consent was obtained before the participation in the study. The study was approved by the London and Surrey borders NHS research and ethics committee.

#### 2.1.2 Study procedure

Simultaneous 12-lead ECG recording using the Mason–Likar 12-lead electrode arrangement and three study S-ICD surface electrodes placed in the conventional S-ICD electrode positions were recorded for three minutes. (See Fig. 1). ECGs were recorded with the participant in a supine position with the head and shoulders raised to approximately 45°. The electrodes used were Ambu Blue Sensor SP single patient use ECG electrodes (Ambu, Denmark), connected to the Porti7 system with ExG shielded carbon cable (1.5 m), microcoax to unipolar snap (TMS international, The Netherlands). ECGs were recorded using a TMSi Porti7 multi-channel signal recorder (TMS international, The Netherlands) attached to a laptop running TMSi polybench v1.30.3.3521 software (TMS international, The Netherlands). The sampling rate was set at 2000 Hz. ECGs were stored in poly5 format.

#### 2.1.3 Randomisation

Participants were allocated to the ‘training dataset’ or to the ‘validation dataset’ using a random number generator. The training dataset was used to generate the transformation coefficients. The validation dataset was then used to test the coefficients.

#### 2.1.4 Selection of representative beat for analysis

The representative QRST complexes that were compared were average beats over each of the recorded ECG leads generated in the following way: QRS complexes were sensed using an R-wave amplitude adaptively decaying sense threshold and any oversensed beats were manually removed. The QRS fiducial point for each patient was aligned for all leads by selecting the median QRS peak sample in time with the first beat in each lead. The starting template was designated to begin 100 milliseconds (ms) prior to the sensed fiducial points and end 450 ms after the sensed fiducial point.

#### 2.1.5 Generation of a signal averaged beat

The correlation coefficient of the template versus each of the following beats was calculated. Initially, only beats with a correlation>0.80 were included in the average. Then, the correlation coefficient was adjusted per lead between 0.5 and 0.9 to ensure the numbers of beats in each lead were similar. In addition, the difference in area between the template and the following beats were assessed for similarity and following beats were excluded if the area was not similar. The resulting signal averaged QRST complex from each patient and lead was used as the “representative” beat for which the transformation coefficients were computed.

#### 2.1.6 Generation of coefficients, conversion matrix and derived ECGs

The transformation coefficients were derived using the least squared difference approach, in which they were optimized for minimum root mean squared (RMS) difference between measured and derived vectors when applied to the training dataset. The optimization was performed in MATLAB (MathWorks, 2014a, Natick, USA) using the optimization toolbox function ‘fmincon’ which is a constrained nonlinear optimization method using the interior-point algorithm. This optimization algorithm is designed to efficiently determine the transformation matrix from the horizontal and vertical leads of the S-ICD to an 8-lead ECG that minimizes the RMSD between measured and computed 8-lead ECGs over all patients and leads.

The following model was created for the matrix calculation from two independent S-ICD vectors to an 8-lead ECG:

leadI=a1×H+b1×V

leadII=a2×H+b2×V

leadV1=a3×H+b3×V

leadV2=a4×H+b4×V

leadV3=a5×H+b5×V

leadV4=a6×H+b6×V

leadV5=a7×H+b7×V

leadV6=a8×H+b8×V

in which H is the horizontal vector, V is the vertical vector and a1…a8 and b1…b8 are the transformation coefficients. Leads III, aVR, aVL, and aVF are redundant [1] and are calculated from known geometries in the Einthoven triangle and therefore these leads were not included in the analysis.

#### 2.1.7 Application of matrix to validation dataset

The measured ECG data for the validation dataset were imported into RashLab (a program for data processing, using the libRasch library (http://www.librasch.org/libRASCH-0.8.35)) where QRS complexes were automatically identified for all beats in the signal. The starting position of the P wave was stored into an array.

Each lead signal was then filtered in MATLAB using a 1st order high pass filter. For each starting position element in the array the next 520 samples were collected to ensure that the entire signal of interest was identified. Once all beats were acquired in this way for each lead, these were averaged for each lead, resampled from 1000 to 256 Hz and stored. This produced an averaged beat for each lead and each patient of 134 samples.

The averaged beats from study leads H and V were then combined with the transformation matrix to generate eight independent derived beats (lead I, II, V1–V6). The derived leads were compared to the measured leads for each patient in RAW format.

Continuous data were presented as mean and standard deviation and categorical variables were presented as frequencies and percentages. The quantitative measures of similarity between the original (measured) ECG and the corresponding derived (reconstructed) ECG were determined using Pearson *r* correlation [2], [3] and root mean square error (RMSE) analysis [3], [4] for each derived lead. The Pearson *r* was considered to show high positive correlation [5] at *r*≥0.7. The RMSE is a parameter that indicates the average voltage error (microvolts) across the ECG leads studied. These parameters have been used by other investigators who have recorded this type of data for derived ECG leads [6]. All analyses were performed in Stata 13 (StataCorp., College Station, TX: StataCorp LP.).

## Figures and Tables

**Table 1:** Summary of RMSE (in microvolts) and Pearson *r* (dimensionless) for all cases.

**Lead**	**RMSE**	**Pearson *r* in µV**
I	292.6	0.585
II	275.1	0.8579
V1	359.58	0.8413
V2	643.93	0.719
V3	638.76	0.6802
V4	618.2	0.5989
V5	455.88	0.7287
V6	242.99	0.8841

**Table 2:** Pearson correlation coefficients (*r*) for all patients and leads.

**Case**	**I**	**II**	**V1**	**V2**	**V3**	**V4**	**V5**	**V6**
1	0.463	0.945	0.984	0.664	0.738	0.817	0.903	0.944
2	0.661	0.985	−0.442	0.771	0.948	0.975	0.980	0.984
3	−0.440	0.847	0.981	0.949	0.949	0.930	0.859	0.554
4	0.966	0.774	0.989	0.992	0.924	0.574	0.692	0.707
5	0.738	0.947	0.960	0.837	0.768	0.597	0.960	0.967
6	0.970	0.988	0.217	−0.223	0.847	0.971	0.987	0.995
7	0.919	0.644	0.996	0.995	0.971	0.575	0.990	0.994
8	0.921	0.982	0.957	0.860	0.636	0.507	0.973	0.997
9	0.607	0.881	0.993	0.964	0.938	0.896	0.866	0.949
10	0.735	0.954	0.992	0.919	0.832	0.862	0.986	0.991
11	0.810	0.944	0.979	0.940	0.935	−0.230	0.975	0.994
12	−0.137	0.849	0.974	0.963	0.949	0.913	0.793	0.288
13	0.944	0.885	0.995	0.990	0.959	0.952	0.974	0.971
14	0.934	0.988	0.918	0.844	0.948	0.954	0.976	0.989
15	0.774	0.969	0.846	0.382	0.865	0.951	0.929	0.943
16	0.905	0.984	0.865	0.152	0.743	0.960	0.982	0.993
17	0.917	0.989	0.985	0.952	0.819	0.754	0.977	0.983
18	0.546	0.948	0.974	0.980	0.995	0.992	0.781	0.595
19	0.968	−0.280	0.969	0.969	0.942	0.866	0.755	0.932
20	0.815	0.885	0.768	0.427	0.637	0.849	0.891	0.924
21	0.809	0.723	0.960	0.962	0.961	0.710	0.779	0.973
22	0.598	0.939	0.922	0.510	0.168	0.507	0.909	0.967
23	0.626	0.963	0.777	0.105	0.347	−0.063	−0.094	0.348
24	0.846	0.977	0.985	0.851	0.721	0.946	0.935	0.966
25	−0.302	0.994	−0.092	−0.376	0.817	0.848	0.903	0.963
26	−0.598	0.991	0.924	0.319	−0.406	−0.617	−0.470	0.971
27	−0.073	0.935	0.926	0.973	0.981	0.973	0.901	0.949

## References

[1] Frank E. (1954). General theory of heart-vector projection. Circ. Res..

[2] Chantad D., Krittayaphong R., Komoltri C. (2006). Derived 12-lead electrocardiogram in the assessment of ST-segment deviation and cardiac rhythm. J. Electrocardiol..

[3] Nelwan S.P., Kors J.A., Crater S.W., Meij S.H., van Dam T.B., Simoons M.L. (2008). Simultaneous comparison of 3 derived 12-lead electrocardiograms with standard electrocardiogram at rest and during percutaneous coronary occlusion. J. Electrocardiol..

[4] Drew B.J., Pelter M.M., Wung S.F., Adams M.G., Taylor C., Evans G.T. (1999). Accuracy of the EASI 12-lead electrocardiogram compared to the standard 12-lead electrocardiogram for diagnosing multiple cardiac abnormalities. J. Electrocardiol..

[5] Munro Barbara Hazard (2005). Correlation. Statistical Methods for Healthcare Research.

[6] Schreck D.M., Fishberg R.D. (2013). Derivation of the 12-lead electrocardiogram and 3-lead vectorcardiogram. Am. J. Emerg. Med..

